# Unexpected Species Identities and Interspecific Relationships in a Subterranean Beetle Lineage, the *Pterostichus macrogenys* Species Group (Coleoptera, Carabidae), Revealed by Fine-Scale Field Sampling and Detailed Morphological Comparisons

**DOI:** 10.3390/insects11110803

**Published:** 2020-11-14

**Authors:** Kôji Sasakawa, Yoshiji Mitsuduka, Hirotarô Itô

**Affiliations:** 1 Laboratory of Zoology, Department of Science Education, Faculty of Education, Chiba University, 1-33 Yayoi-cho, Inage-ku, Chiba-shi, Chiba 263-8522, Japan; 2 2-18-24 Hanatate Yamagata-shi, Yamagata 990-0067, Japan; mushiking4@yahoo.co.jp; 3 1-14-16 Awayama, Niigata-shi, Niigata 950-0843, Japan; piropirod2ihold@gmail.com

**Keywords:** body size, endophallus, Japan, male genitalia, new distribution records, new species, species coexistence, sympatric occurrence

## Abstract

**Simple Summary:**

Morphological and ecological features are highly specialized among subterranean insect species. Studies of such features reveal the vast diversity of insect taxa and can also provide insight into the general mechanisms associated with species diversity. The *Pterostichus macrogenys* species group is an endemic subterranean Japanese carabid beetle lineage that shows marked regional differentiation. However, to date, its diversity has not been fully elucidated, partly due to the difficulty of sample collection. We examined 103 specimens from this species group, which were collected by fine-scale field sampling, and classified these into one new and eight known species. The results of this study revealed that some of these species have disjunct distributions, which have not been reported in this species group. More importantly, some species of this group coexisted in some localities; in most such cases, two species with different body sizes coexisted, implying a role of differential body size in promoting coexistence. In the remaining case, one large and two small species coexisted, and the two small species have male genitalia of different sizes; in this system, body and genital size differences appear to have different effects on coexistence, implying a species coexistence mechanism that has rarely been reported in insects.

**Abstract:**

An endemic subterranean Japanese carabid beetle lineage, the *Pterostichus macrogenys* species group, was recently revealed to have marked regional differentiation. Studies of such features reveal insect species diversity and provide insight into the mechanisms driving species diversity. We examined specimens of this species group collected from the southern Tohoku District of Honshu, Japan, where its diversity has not yet been fully elucidated, using fine-scale field sampling and detailed comparative morphological analysis of male genitalia. In total, 103 specimens from 13 localities were classified into one new (*P. monolineatus* sp. n.) and eight known species. In four of the known species, we observed disjunct distributions, which have not previously been reported in this species group and may be more common than previously recognized. Species coexistence was observed at four sites, with two species of different body sizes coexisting at three sites and three species coexisting at the remaining site. The three coexisting species included one large and two small species, the latter of which have male genitalia of a different size. This newly discovered coexistence pattern implies separate effects of differential body and genital size in species coexistence, which has rarely been reported in insects.

## 1. Introduction

Morphological and ecological features are highly specialized among subterranean insects [1]. These features have been the subject of many taxonomic and evolutionary studies [2,3,4]. Studies of subterranean insects can provide insights into the mechanisms underlying interspecific relationships [5], which are considerably influenced by various abiotic and biotic factors in aboveground systems, complicating the interpretation of results. In contrast, subterranean systems generally comprise a limited range of environments [5,6,7]. Consequently, the interspecific relationships observed in subterranean environments can be considered to be primarily influenced by biotic rather than abiotic factors, simplifying evaluation of the effects of biotic factors [5]. However, to date, few studies of subterranean insects have been conducted, mainly because of difficulties in sample collection [6] and analysis associated with the rarity and small size of specimens [8]. Therefore, further studies are needed.

The *macrogenys* species group is a clade of the carabid beetle *Pterostichus* subgenus *Nialoe* Tanaka, 1958 [9,10,11]. This flightless group was previously considered to consist of *P. macrogenys*, 1883, which is widely distributed from central to northern Honshu, Japan, and one or two other species with limited distribution [12,13,14,15]. This species classification system was based on a lack of definitive differences in the shape of the aedeagus in the male genitalia, which has been used as an important taxonomic character in *Nialoe*, among a small number of specimens from several localities [13]. Later, it became apparent that members of this species group occur mainly in subterranean environments and can be effectively collected using subterranean baited traps; subsequently, many specimens have been collected [10,16]. The shape of the endophallus (a membranous inner sac everted from the aedeagus), which had not previously been examined in the taxonomy of this group, was eventually found to be useful for classification [17,18]. With the development of sampling and examination techniques, the species diversity of the *macrogenys* species group has been elucidated over the past 15 years, and now includes more than 30 species [11]. In most regions, only one species has been found, whereas in some localities, two species have been found to be sympatric [10,16]. However, there are many localities from which no specimens have been examined; therefore, specimens from these localities are badly needed [18].

**Figure 1:**
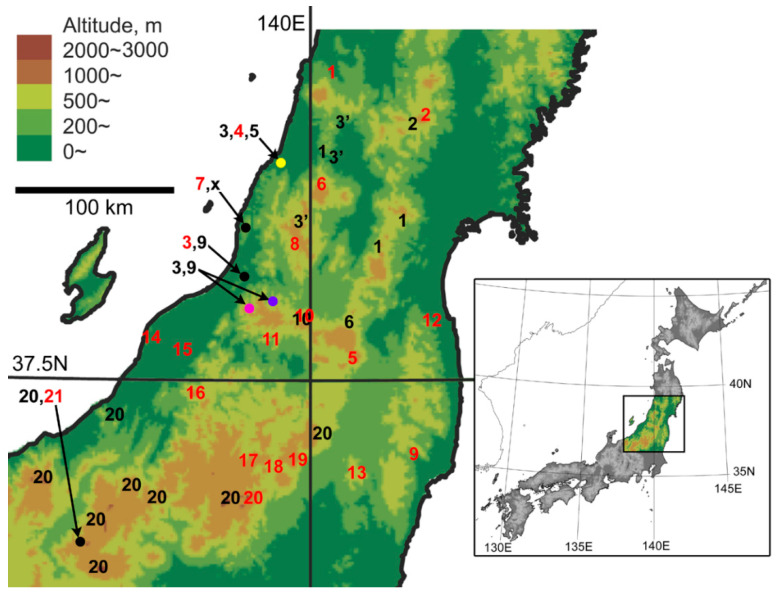
Distribution of the *Pterostichus macrogenys* species group in northern Chubu, northern Kanto, and southern Tohoku Districts of Honshu, Japan. (**1)** *P. chokaisanus* Sasakawa, 2009; (**2**) *P. kurikomasanus* Sasakawa, 2005; (**3**) *P. falcispinus* Sasakawa, 2005 “western type”; (**3′**) *P. falcispinus* “eastern type”; (**4**) *P. takadateyamanus* Sasakawa, 2009; (**5**) *P. adatarasanus* Sasakawa, 2005; (**6**) *P. gassanus* Sasakawa, 2009; (**7**) *P. shinbodakensis* Sasakawa and Itô, 2017; (**8**) *P. asahinus* Habu and Baba, 1960; (**9**) *P. iwakiensis* Sasakawa, 2009; (**10**) *P. eboshiyamanus* Sasakawa, 2009; (**11**) *P. tateishiyamanus* Sasakawa and Itô, 2017; (**12**) *P. monolineatus* sp. n.; (**13**) *P. yamizosanus* Sasakawa, 2005; (**14**) *P. yahikosanus* Sasakawa, 2009; (**15**) *P. ohsawacavus* Sasakawa, 2005; (**16**) *P. sumondakensis* Sasakawa, 2005; (**17**) *P. isolatus* Sasakawa, 2005; (**18**) *P.* *nakamiyorinus* Morita, Ohkawa and Kurihara, 2013; (**19**) *P. momuranus* Morita, Ohkawa and Kurihara, 2013; (**20**) *P. macrogenys* Bates, 1883; (**21**) *P. nagasawai* Ito and Ogai, 2015; **x** unidentified species sympatric with *P. shinbodakensis*. Red letters indicate the type localities of each species.

This study reports our findings on the *macrogenys* species group collected from the southern Tohoku District, based on fine-scale field sampling and analysis of about 100 specimens from a wide area within the district (Figure 1). Our results will provide insights into the mechanisms underlying sympatric occurrence of these closely related species.

## 2. Materials and Methods

In this study, most specimens were collected using subterranean baited traps (STs). The STs used by Y. Mitsuduka (YM) were constructed according to the methods of Yoshida [19] using silkworm pupal powder as an attractant and Galt’s solution as a preservative. The structure, attractant (silkworm pupal powder), and preservative (10% acetic acid) of STs used by H. Itô (HI) were as described by Sasakawa and Itô [10]. Some specimens were collected using conventional (i.e., at the soil surface) pitfall traps (PTs). Specimens examined are deposited in the collection of the National Museum of Nature and Science, Tsukuba, Japan (NSMT) and in the authors’ collections.

The identification of male specimens was based mainly on comparisons of endophallus morphology. The endophallus was everted by injecting toothpaste at the basal end of the aedeagus [20] and compared with photographs of the endophalli of holotypes of all species that are distributed at or near our collection sites. The holotype photographs were taken during our previous study [10,17,18] and were sufficient for species identification in the present study. The endophallus structural terminology used in this study follows that of Sasakawa [18]. Female specimen identification was based on comparisons of the morphological and morphometric features of conspecific male specimens that were obtained at or near the same collection site and identified from endophallus morphology. Thus, female specimens for which conspecific male specimens were unavailable could not be identified.

Within the *macrogenys* species group, the body sizes of observed and presumed sympatric species have been reported to differ significantly [10,18], implying that body size is a key trait associated with species coexistence. Therefore, in this study, we evaluated body size in our specimens using three body length measurements: mandible apices to elytral end (BLm), anterior margin of labrum to elytral end (BLl), and clypeal apex to elytral end (BLc) [10,18]. Measurements were obtained using ImageJ software ver. 1.50i [21], based on dorsal view photographs taken using a digital camera.

## 3. Results

### 3.1. Overview of Species Identification, Distribution, and Coexistence

**Figure 2:**
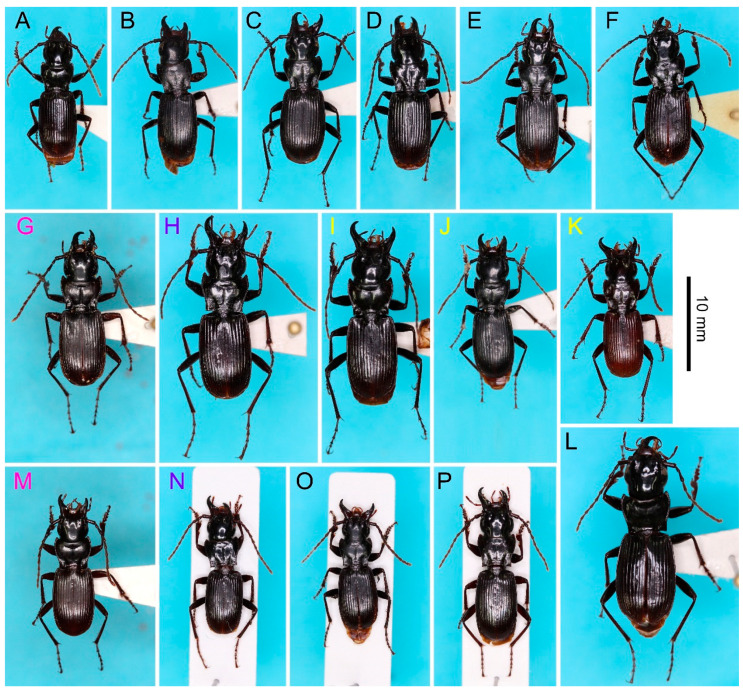
Habitus dorsal view of the *macrogenys* species group; (**A**), (**B**) *P. chokaisanus* males from Sakunami (**A**) and Kamihôzawa (**B**); (**C**) *P. kurikomasanus* male from Yuhama Pass; (**D**–**I**) *P. falcispinus* males from Kurosawagawa forest road (**D**), Togawa (**E**), Ôtori (**F**), Miyahisa (**G**), Nukumidaira (**H**), and Mt. Takadateyama (**I**), (**J**) *P. takadateyamanus* male from Mt. Takadateyama; (**K**) *P. adatarasanus* male from Mt. Takadateyama; (**L**) *P. gassanus* male from Goshiki forest road; (**M**–**N**) *P. iwakiensis* males from Miyahisa (**M**) and Nukumidaira (**N**); (**O**) *P. eboshiyamanus* male from Yunosawa; (**P**) *P. monolineatus* sp. n. holotype male from Aobaminami. Pink, purple, and yellow letters indicate that specimens were obtained at collection sites indicated by these respective colors in Figure 1.

**Figure 3:**
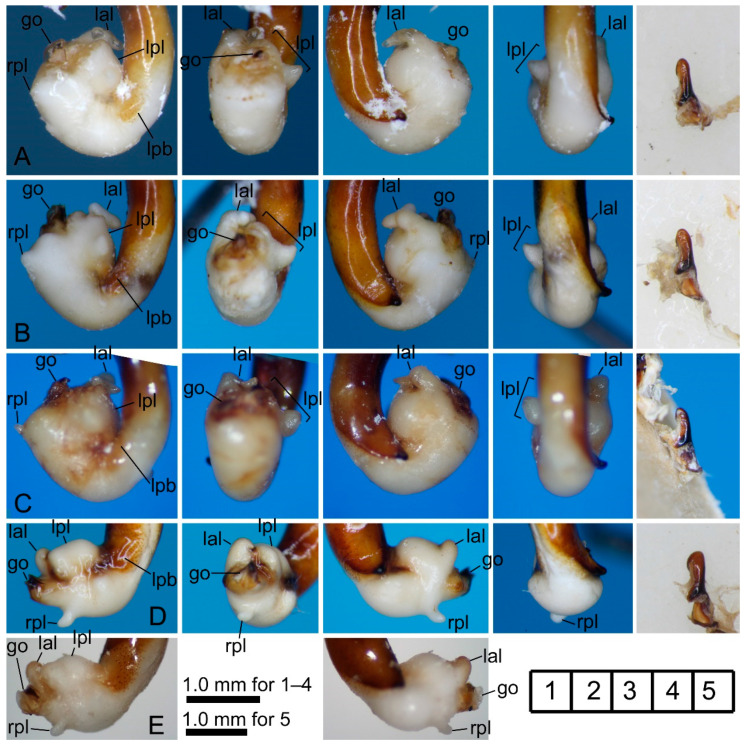
Left lateral (**1**), ventral (**2**), right lateral (**3**), and dorsal (**4**) views of aedeagus (part) with the everted endophallus and left lateral view (**5**) of right paramere of the *macrogenys* species group; (**A**–**C**) *P. chokaisanus* males from Sakunami (**A**), Kamihôzawa (**B**), and holotype male from “Yashima” (**C**), (**D**,**E**) *P. kurikomasanus* male from Yuhama Pass (**D**) and holotype male from “Mts Kurikomasan” (**E**). go: gonopore, lal: left apical lobe, lpl: left preapical lobe, rpl: right preapical lobe. Numbers in the grid on the right bottom indicate subfigure placement.

**Figure 4:**
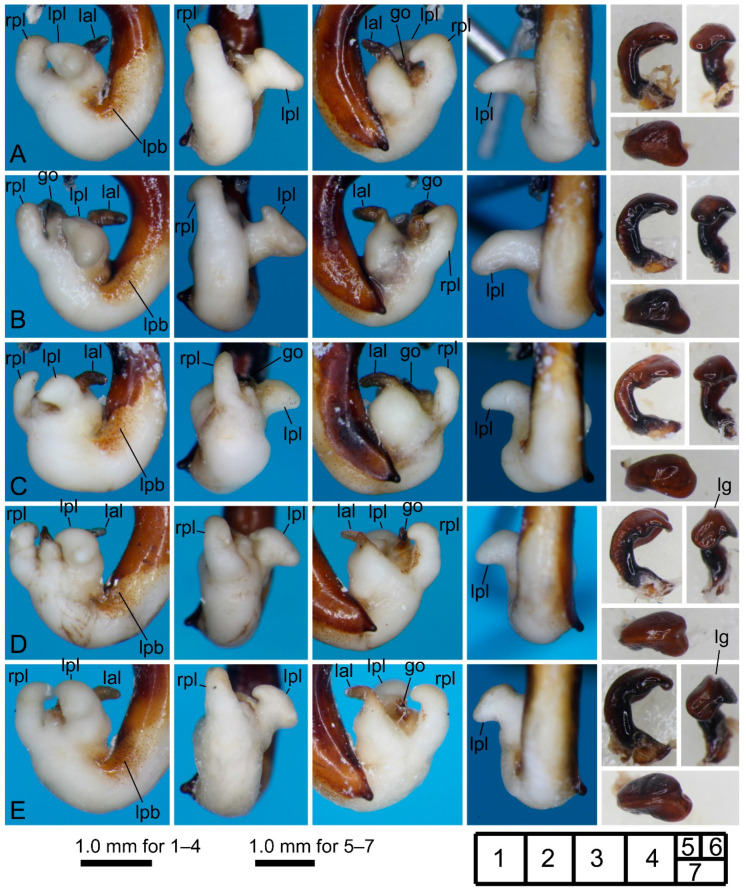
Left lateral (**1**), ventral (**2**), right lateral (**3**), and dorsal (**4**) views of aedeagus (part) with the everted endophallus and left lateral (**5**), apical (**6**), and dorsal (**7**) views of right paramere of *Pterostichus falcispinus* males from Kurosawagawa forest road (**A**), Togawa (**B**), Ôtori (**C**), Miyahisa (**D**), and Nukumidaira (**E**). lg: longitudinal groove.

**Figure 5:**
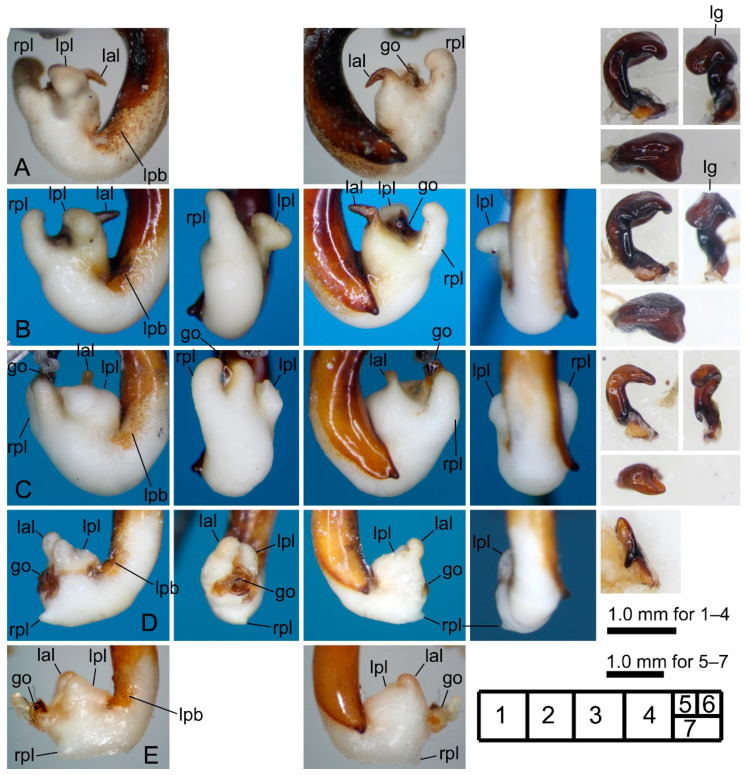
Left lateral (**1**), ventral (**2**), right lateral (**3**), and dorsal (**4**) views of aedeagus (part) with the everted endophallus and left lateral (**5**), apical (**6**), and dorsal (**7**) views of right paramere of the *macrogenys* species group; (**A**,**B**) *P. falcispinus* males from type locality (**A1, 3** holotype male from “Cave Ishikiri”, **A5**–**7** a non-type male from Mt. Ishikiriyama) and Mt. Takadateyama (**B**), (**C**) *P. takadateyamanus* male from Mt. Takadateyama, (**D**,**E**) *P. adatarasanus* male from Mt. Takadateyama (**D**) and holotype male from “Tsuchiyu-Pass” (**E**).

**Figure 6:**
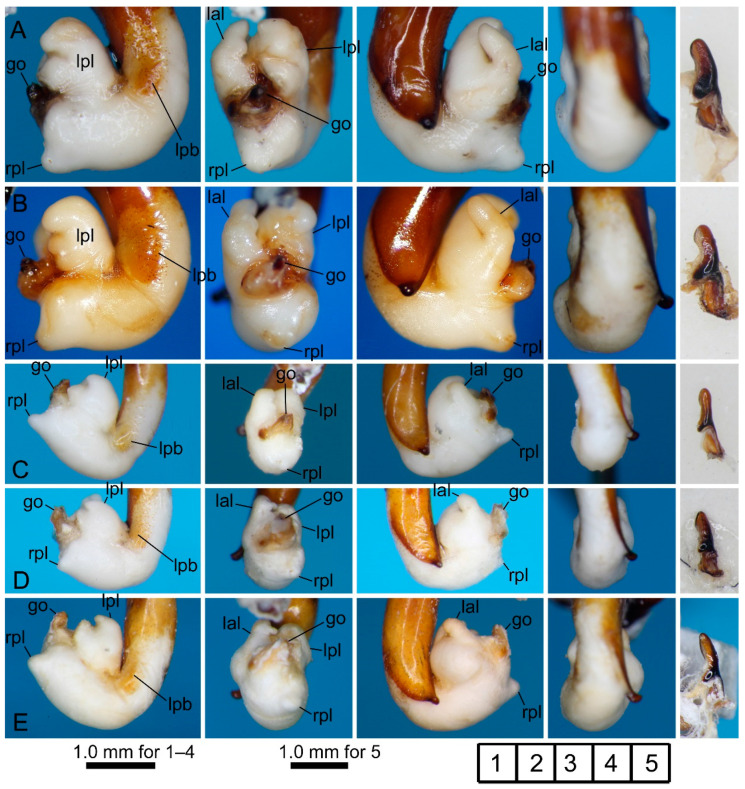
Left lateral (**1**), ventral (**2**), right lateral (**3**), and dorsal (**4**) views of aedeagus (part) with the everted endophallus and left lateral view (**5**) of right paramere of the *macrogenys* species group; (**A**,**B**) *P. gassanus* male from Goshiki forest road (**A**) and holotype male from “Riv. Tachiyazawagawa” (**B**), (**C**–**E**) *P. iwakiensis* males from Miyahisa (**C**) and Nukumidaira (**D**) and holotype male from “Iritôno” (**E**).

**Figure 7:**
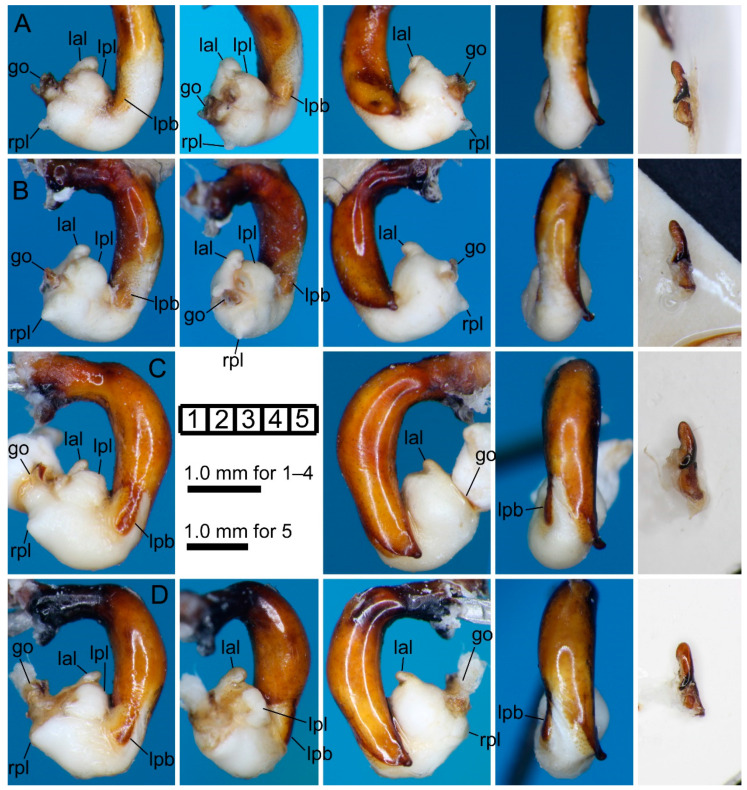
Left lateral (**1**), ventral (**2**), right lateral (**3**), and dorsal (**4**) views of aedeagus (part) with the everted endophallus and left lateral view (**5**) of right paramere of the *macrogenys* species group; (**A**,**B**) *P. eboshiyamanus* male from Yunosawa (**A**) and holotype male from “River Tokorozawa” (**B**), (**C**,**D**) *P. monolineatus* sp. n. holotype (**C**) and paratype (**D**) male from Aobaminami.

**Figure 8:**
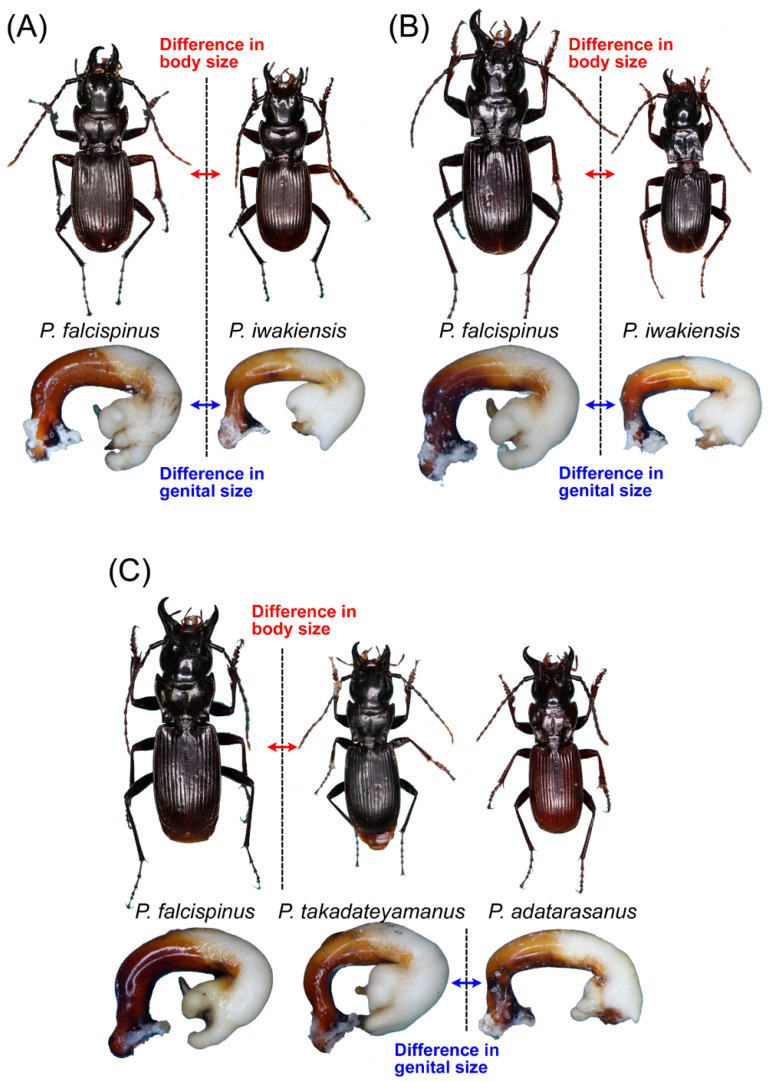
Body size and male genitalia size differences among sympatric species at Miyahisa (**A**), Nukumidaira (**B**), and Mt. Takadateyama (**C**).

In total, we obtained 118 specimens from 15 localities. Among these, 103 specimens from 13 localities were identified based on their own endophallus morphology or endophallus morphology of putative conspecific males. The 103 specimens were classified into nine species consisting of one new species and eight known species: *P. chokaisanus* Sasakawa, 2009; *P. kurikomasanus* Sasakawa, 2005; *P. falcispinus* Sasakawa, 2005; *P. takadateyamanus* Sasakawa, 2009; *P. adatarasanus* Sasakawa, 2005; *P. gassanus* Sasakawa, 2009; *P. iwakiensis* Sasakawa, 2009; *P. eboshiyamanus* Sasakawa, 2009 (Figure 2, Figure 3, Figure 4, Figure 5, Figure 6 and Figure 7). Among these, all collection records, except for *P. takadateyamanus* and *P. falcispinus* from their type localities, represent new distribution records. For *P. chokaisanus*, *P. adatarasanus*, *P. gassanus*, and *P. iwakiensis*, the collection sites of this and previous studies were discontinuous, reciprocally distant mountains with large distributional gaps, representing typical disjunct distribution (Figure 1). The collection sites of *P. falcispinus* were also several discontinuous mountains; however, their reciprocal distances were shorter than those of the other four species, representing typical wide distribution, rather than disjunct distribution (Figure 1). Species coexistence was confirmed at four collection sites (Figure 1 and Figure 8): (i) *P. falcispinus*, *P. takadateyamanus*, *P. adatarasanus* at Mt. Takadateyama; (ii) *P. falcispinus* and *P. iwakiensis* at Miyahisa; (iii) *P. falcispinus* and *P. iwakiensis* at Nukumidaira; (iv) *P. falcispinus* and *P. iwakiensis* at Mt. Ishikiriyama. In the following section, detailed information on the collected specimens is reported with comments, and a description of the new species is provided. The Japanese names of all species examined in this study are provided in Table S1.

### 3.2. Collection Records and Systematics

#### 3.2.1. *Pterostichus (Nialoe) chokaisanus* Sasakawa, 2009

Figure 2A,B and Figure 3A–C.

Materials examined. 3♂1♀, Sakunami, alt. 485 m, Sendai-shi, Miyagi Prefecture, 15. vi–24. vii. 2017, ST, leg. YM; 2♂1♀, Kamihôzawa, alt. 493 m, Yamagata-shi, Yamagata Prefecture, 10. vi–3. vii. 2016, ST, leg. YM (1♂, 10. vi–3. vii. 2016; 1♂, 12. vii–18. viii. 2017; 1♀, 18. viii–12. ix. 2017).

Body length (mean ± SD (mm)). ♂ (*n* = 5), BLm 13.95 ± 0.72, BLl 12.84 ± 0.44, Blc 12.41 ± 0.43; ♀ (*n* = 2), BLm 14.49 ± 0.37, BLl 13.21 ± 0.06, Blc 12.71 ± 0.1.

Notes. The endophalli of the Sakunami and Kamihôzawa specimens differ from that of the holotype from “Yashima” [18] in that the left preapical lobe is conical in dorsal view (in holotype, ellipsoid rather than conical); however, this morphological difference was judged to be not of species level. This is the first record of a female specimen.

#### 3.2.2. *Pterostichus (Nialoe) kurikomasanus* Sasakawa, 2005

Figure 2C and Figure 3D,E.

*Material examined.* 1♂, Yuhama Pass, 254 m, Hanayama, Kurihara-shi, Miyagi Prefecture, 2. vii–25. viii. 2016, ST, leg. YM.

Body length (mean ± SD (mm)). ♂ (*n* = 1), BLm 15.62, BLl 14.15, Blc 13.58.

Notes. No conspicuous difference in endophallus structure between the holotype from “Mts. Kurikomasan” [17] and the Yuhama specimen.

#### 3.2.3. *Pterostichus (Nialoe) falcispinus* Sasakawa, 2005

Figure 2D–I, Figure 4A–E and Figure 5A,B.

Materials examined. 4♂2♀, Kurosawagawa forest road, alt. 254 m, Takasaka, Mamurogawa-machi, Yamagata Prefecture, ST, leg. YM (2♂, 14. vii–15. viii. 2016; 1♂1♀, 11. vi–5. vii. 2017; 1♀, 5. vii–21. viii. 2017; 1♂, 7. vii–8. viii. 2018); 3♂, Togawa, alt. 47 m, Furukuchi, Tozawa-mura, Yamagata Prefecture, 14. vi–29. vii. 2017, ST, leg. YM; 2♂1♀, Ôtori, alt. 405 m, Tsuruoka-shi, Yamagata Prefecture, 26. vii–7. ix. 2017, ST, leg. YM; 1♂, Miyahisa, alt. 262 m, Tainai-shi, Niigata Prefecture, 26–27. ix. 2020, PT, leg. H. Itô; 4♂10♀, Nukumidaira, alt. 458 m, Kotamagawa, Oguni-machi, Yamagata Prefecture, ST, leg. YM (2♂6♀, 1–18. ix. 2015; 2♂1♀, 2–17. x. 2015; 1♀, 9–29. viii. 2016; 2♀, 31. vii–6. ix. 2017); 1♂3♀, Mt. Takadateyama, alt. 62 m, Tsuruoka-shi, Yamagata Prefecture, ST, leg. YM (1♂2♀, 17. v–2. vi. 2017; 1♀, 4–31. vii. 2018); 1♂1♀, Mt. Ishikiriyama, Haguro, Tainai-shi, Niigata Prefecture, leg. HI (1♂, dark zone of Ishikiri Cave, alt. 149 m, 19–21. v. 2013, PT; 1♀, entrance of Ishikiri Cave, alt. 163 m, 6–21. iii. 2016, ST).

Body length (mean ± SD (mm)). Values are given for two geographical types with distinctly different body sizes, which are defined in the Notes. Western type (Mt. Takadateyama, Mt. Ishikiriyama, Miyahisa, and Nukumidaira populations): ♂ (*n* = 7), BLm 17.75 ± 1.1, BLl 15.79 ± 0.86, Blc 15.23 ± 0.81; ♀ (*n* = 14), BLm 18.92 ± 0.95, BLl 16.79 ± 0.76, Blc 16.22 ± 0.7. Eastern type (Kurosawagawa, Togawa, and Ôtori populations): ♂ (*n* = 9), BLm 15.31 ± 0.85, BLl 13.96 ± 0.63, Blc 13.49 ± 0.59; ♀ (*n* = 3), BLm 15.53 ± 0.61, BLl 13.98 ± 0.4, Blc 13.52 ± 0.44.

Notes. Some differences in the characteristics of the endophallus (e.g., shape/size of lobes on the surface) were recognized among our male specimens. Whether these differences are due to geographical or individual variation or specimen preparation remains unknown; however, all had the same basic endophallus structure as the *P. falcispinus* holotype from “Cave Ishikiri” [17], which is a cave at Mt. Ishikiriyama. Therefore, all male specimens were identified as *P. falcispinus*, although this result requires further study as discussed later. The identification of female specimens was based on the characteristics of the pronotum (cordate shape and pronounced anterior angles) and body size.

Although geographical variation in endophallus morphology remains unclear, two conspicuous geographic variants were recognized based on the shape of the right paramere and body size. The western type consists of populations from Mt. Takadateyama, Mt. Ishikiriyama, Miyahisa, and Nukumidaira, and is characterized by a longitudinal groove on the dorsum of the right paramere and, except for the Miyahisa population, by a larger body, although smaller body size requires further confirmation for the Miyahisa population (BLm, 16.10; BLl, 14.53; BLc, 14.01) because only one specimen was available from this locality. The eastern type is characterized by the absence of a groove on the dorsum of the right paramere and by a smaller body, and consists of populations from Kurosawagawa, Togawa, and Ôtori. The groove on the dorsum of the right paramere was not found in other *Nialoe* species and is an unambiguously apomorphic character. Apomorphy and larger body size (in Carabidae, body size is generally larger in derived clades [22]) indicate that the western type is more derived than the eastern type.

Unexpectedly, we discovered that *P. asahinus*, which was described based on a single female from Mt. Dorokujinpô and whose male is unknown, and *P. falcispinus* may be conspecific. The reasons for this conclusion are that (i) the type locality of *P. asahinus* is surrounded by our collection sites for *P. falcispinus* (Figure 1), and (ii) *P. asahinus* and some *P. falcispinus* populations have almost the same body size (*P. asahinus*: BLm, 15.2 [12], similar to those of the eastern type and the Miyashita population of the western type). If the eastern type of *P. falcispinus* is demonstrated to be identical to *P. asahinus*, then the name *asahinus* is applied to the eastern type, although its taxonomic status (species or subspecies) remains unresolved. If the western type is demonstrated to be identical to *P. asahinus*, then *P. falcispinus* is synonymized with *P. asahinus*, although the taxonomic treatment of the eastern type remains unresolved. Further examination of male *P. asahinus* specimens from its type locality is necessary to resolve these taxonomic problems.

#### 3.2.4. *Pterostichus (Nialoe) takadateyamanus* Sasakawa, 2009

Figure 2J and Figure 5C.

Materials examined. 2♂3♀, Ôyama, near Ôyamakamiike pond, alt. 62 m, the southern foot of Mt. Takadateyama, Tsuruoka-shi, Yamagata Prefecture, ST, leg. YM (1♀, 16. vi–5. vii. 2016; 2♂2♀, 31. vii–30. viii. 2018); 11♂21♀, Ôyama, alt. 232 m, near the summit of Mt. Takadateyama, Tsuruoka-shi, Yamagata Prefecture, ST, leg. HI (10♂19♀, 22. v–22. vi. 2014; 1♂2♀, 25. iv–24. v. 2015); 1♀, Kanazawa, Mt. Takadateyama, Tsuruoka-shi, Yamagata Prefecture, 7. vii–8. viii. 2018, ST, leg. YM.

Body length (mean ± SD (mm)). ♂ (*n* = 13), BLm 14.54 ± 0.78, BLl 13.24 ± 0.66, Blc 12.74 ± 0.65; ♀ (*n* = 25), BLm 15.06 ± 0.88, BLl 13.56 ± 0.71, Blc 13.04 ± 0.69.

Notes. Currently, this species is known only from the type locality “Mt. Takadate” [18]. The identification of female specimens was based on body size and two characteristics of the pronotum (cordate shape and pronounced anterior angles), by which conspecific males can be readily distinguished from males of similar-sized, sympatric *P. adatarasanus*.

#### 3.2.5. *Pterostichus (Nialoe) adatarasanus* Sasakawa, 2005

Figure 2K and Figure 5D,E.

Materials examined. 2♂3♀, Ôyama, near Ôyamakamiike pond, alt. 62 m, the southern foot of Mt. Takadateyama, Tsuruoka-shi, Yamagata Prefecture, 4–31. vii. 2018, ST, leg. YM.

Body length (mean ± SD (mm)). ♂ (*n* = 2), BLm 14.66 ± 0.02, BLl 13.23 ± 0.05, Blc 12.79 ± 0.06; ♀ (*n* = 3), BLm 15.61 ± 0.28, BLl 13.94 ± 0.3, Blc 13.5 ± 0.26.

Notes. Compared to the holotype endophallus, that of the Takadateyama specimens has a slightly longer left apical lobe and slightly slenderer right preapical lobe; however, these differences were judged not to be of species level. The identification of female specimens was based on body size and on two characteristics of the pronotum (weakly cordate shape and less pronounced anterior angles), by which conspecific males can be readily distinguished from males of *P. takadateyamanus*, which are sympatric at Mt. Takadateyama and have similar body size. This is the first record of a female specimen.

#### 3.2.6. *Pterostichus (Nialoe) gassanus* Sasakawa, 2009

Figure 2L and Figure 6A,B.

Materials examined. 2♂, Goshiki forest road, alt. 809 m, Ôsawa, Yonezawa-shi, Yamagata Prefecture, ST, leg. YM (1♂, 26. v–7. vii. 2015; 1♂, 3–18. ix. 2015).

Body length (mean ± SD (mm)). ♂ (*n* = 2), BLm 19.75 ± 0.23, BLl 18.1 ± 0.22, Blc 17.46 ± 0.14.

Notes. The Goshiki specimens and the holotype have slightly different characteristics at the basal half of the left preapical lobe of the endophallus; however, this difference was judged to be not of species level.

#### 3.2.7. *Pterostichus (Nialoe) iwakiensis* Sasakawa, 2009

Figure 2M,N and Figure 6C–E.

Materials examined. 3♂1♀, Miyahisa, alt. 262 m, Tainai-shi, Niigata Prefecture, PT, leg. HI (1♂1♀, 19–20. ix. 2020; 2♂, 26–27. ix. 2020); 1♂4♀, Nukumidaira, alt. 458 m, Kotamagawa, Oguni-machi, Yamagata Prefecture, ST, leg. YM (1♂1♀, 1–18. ix. 2015; 1♀, 9–29. viii. 2016; 2♀, 31. vii–6. ix. 2017); 1♀, dark zone of Ishikiri Cave, Mt. Ishikiriyama, alt. 163 m, Haguro, Tainai-shi, Niigata Prefecture, 23. ix–1. x. 2017, PT, leg. HI.

Body length (mean ± SD (mm)). ♂ (*n* = 4), BLm 14.1 ± 0.7, BLl 12.68 ± 0.61, Blc 12.25 ± 0.6; ♀ (*n* = 6), BLm 14.32 ± 0.58, BLl 13.01 ± 0.53, Blc 12.55 ± 0.51.

Notes. Compared to type materials from “Iritôno” [18], our specimens have a more weakly swollen left lateral side of the endophallus, less convex pronotum, and pronotal laterobasal impressions extending more anteriorly; however, these differences were judged to be not of species level. The identification of female specimens, particularly the specimen from Mt. Ishikiriyama where male specimens have been unavailable, was based on body size and pronotum characteristics.

#### 3.2.8. *Pterostichus (Nialoe) eboshiyamanus* Sasakawa, 2009

Figure 2O and Figure 7A,B.

Materials examined. 1♂3♀, Yunosawa, alt. 820 m, Hirogawara, Iide-machi, Yamagata Prefecture, ST, leg. YM (1♀, 2–17. x. 2015; 1♂2♀, 2. vii–19. viii. 2018).

Body length (mean ± SD (mm)). ♂ (*n* = 1), BLm 13.7, BLl 12.32, Blc 11.82; ♀ (*n* = 3), BLm 13.84 ± 0.57, BLl 12.64 ± 0.66, Blc 12.2 ± 0.69.

Notes. We found no conspicuous difference in endophallus structure between the holotype from “River Tokorozawa on the western slope of the Eboshiyama Hills” [18] and the Yunosawa specimen. This is the first record of a female specimen.

#### 3.2.9. *Pterostichus (Nialoe) monolineatus* sp. n.

Figure 2P and Figure 7C,D.

Type materials. Holotype: ♂, Aobaminami, alt. 310 m, Ôuchi, Marumori-machi, Miyagi Prefecture, 10. ix–4. x. 2019, ST, leg. YM; paratypes: 1♂1♀, same data as the holotype. All type materials are to be deposited in the collection of the NSMT.

Diagnosis. This species is readily distinguished from all members of the *macrogenys* species group by the convex middle dorsal side of the aedeagus and a clearly band-shaped, strongly sclerotized left pigmented band.

Body length (mean ± SD (mm)). ♂ (*n* = 2), BLm 15.17 ± 1.37, BLl 13.87 ± 0.88, Blc 13.39 ± 0.83; ♀ (*n* = 1), BLm 15.5, BLl 14.01, Blc 13.43.

Description. Dorsal surface of body blackish, shiny, not opaque; surface almost smooth, except for laterobasal impressions of the pronotum, which are slightly punctated. Appendages dark brown to blackish.

Head large, widest at tempora, which are distinctly swollen; width at the widest point larger than pronotal posterior margin width; length from clypeal apex to neck base larger than pronotum length along the median line. Left mandible large and curved at the apical 1/4; linear length between apex and posterolateral end of left mandible ~3.0-fold as long as the anterior width of the clypeus. Eyes weakly convex, with the anterior–posterior length longer than 1/2 of the length of antennal segment 1. Antennal segment 2 with two setae.

Pronotum cordate notably flat, widest at apical 1/4 in male and 1/5 in female. Lateral margins arcuate on apical 2/3, slightly sinuate on basal 1/3; two marginal setae on each lateral side, anterior setae near widest pronotal point and posterior setae near hind angles. Anterior margin slightly bisinuate; anterior angles notably pronounced in females, moderately pronounced in males. Posterior margin emarginated at median area, almost straight at lateral areas; hind angles right-angled. Median line impressed in the middle, not reaching both anterior and posterior margins; laterobasal impressions single, shallow.

Elytra almost parallel, less convex; shoulder distinct, but not denticulate; apices rounded, not denticulate; scutellar stria absent in the holotype male, present but not connected to stria 1 in the male and female paratypes; one setigerous puncture on interval 1 near stria 2 at the level of the posterior end of the scutellum; two setigerous punctures on interval 3, with the anterior puncture slightly in front of the middle and posterior punctures on apical 1/5, both adjoining stria 2. Hind wings completely atrophied. Male sternum 7 slightly concave, without conspicuous sexual characteristics. Female first fore tarsomere without adhesive hairs on ventral side.

Aedeagus stout, bent at basal 1/3, convex on dorsal surface at the middle. Endophallus short, stout, strongly bent ventrally, with gonopore directed backward; left pigmented band clearly band-shaped, sclerotized at the same level as the aedeagus, and 1/4–1/3 the width of the adeagus at that position; right preapical lobe rudimentary and only slightly swollen; left preapical lobe moderate in size, with apex not bifurcated and left apical lobe small. Left paramere square. Right paramere short, straight, apically rounded.

Notes. Judging from the similarity of the endophallus structure, this new species is likely most closely related to *P. eboshiyamanus* among the known members of the *macrogenys* species group.

## 4. Discussion

The present study revealed a number of findings on the taxonomy of the *macrogenys* species group. These findings are primarily based on comparative morphological analysis of the endophallus, reaffirming the importance of this male genital morphological feature in the taxonomical classification of this group. We also found that disjunct distributions are more common within the *macrogenys* species group than previously recognized. This finding is noteworthy because, despite insufficient examinations of endophallus morphology, some previous studies have readily described new species when the specimen collection site was somewhat distant from those of other known species, based on slight morphological differences in non-endophallus characters [23,24,25,26,27,28,29,30]. Therefore, some of these species may actually be conspecifics; future studies should resolve this issue by examining the detailed endophallus morphology of these specimens, as in the present study.

The results of the present study enhance our understanding of the general mechanisms of coexistence among closely related species. In the *macrogenys* species group, sympatric occurrence has been reported in two species pairs: *P. nagasawai* Ito and Ogai, 2015 and *P. macrogenys* [16], and *P. shinbodakensis* Sasakawa and Itô, 2017 and its sympatric unidentified species [10]. In the latter species pair, a body size difference was reported, suggesting a role of body size in species coexistence. The present study reports two additional cases of species coexistence: *P. falcispinus* and *P. iwakiensis* at or near the Iidesanchi Mountains (Miyahisa, Nukumidaira, and Mt. Ishikiriyama); *P. falcispinus*, *P. takadateyamanus*, and *P. adatarasanus* at Mt. Takadateyama (Figure 8). Among these, differences in body size were observed between the two species at the Iidesanchi Mountains and between *P. falcispinus* and the other two species at Mt. Takadateyama, although the size difference between the Ishikiri Cave pair was less clear than those of the other pairs. However, no conspicuous difference in body size was observed in *P. takadateyamanus* and *P. adatarasanus* at Mt. Takadateyama; instead, a definitive difference in the size of the male genitalia was observed between these two species. This pattern implies that differences in genital size, not body size, play an important role in the sympatric occurrence of *P. takadateyamanus* and *P. adatarasanus* at Mt. Takadateyama. Consequently, at Mt. Takadateyama, body and genital size differences appear to have separate effects on species coexistence. To our knowledge, this phenomenon has been reported only in the flightless carabid beetle *Carabus* subgenus *Ohomopterus* Reitter, 1896, which consists of many species with limited distribution, as well as the *macrogenys* species group. In *Ohomopterus*, several species with different body sizes coexist in most areas of the distribution. However, on one mountain (Mt. Kongôsan), two species with similar body sizes but significantly different genital sizes (*C. iwawakianus* (Nakane, 1953) and *C. uenoi* (Ishikawa, 1960)) coexist, such that this mountain provides habitat for the greatest number of *Ohomopterus* species occurring in a single local area [31,32]. Similar findings for phylogenetically distant-related groups (*Pterostichus* in subfamily Harpalinae and *Carabus* in subfamily Carabinae) may indicate that a separate effect due to differences in body and genital size is an important mechanism for the coexistence of closely related species. As with *Ohomopterus*, future studies of the *macrogenys* species group also need to quantitatively evaluate differences in body and genital size among species and examine the evolutionary process of the size differences [32,33].

## 5. Conclusions

Based on fine-scale field sampling and detailed comparative morphological analysis of male genitalia, this study gained new insight into the diversity of the *Pterostichus macrogenys* species group in the southern Tohoku District of Honshu, Japan. One new species (*Pterostichus monolineatus* sp. n.) is described, and eight known species are recorded. The collection records include disjunct distributions of some species and sympatric occurrence of closely related species at some localities. Both body size differences and genital size differences appear to contribute to species coexistence.

## Supplementary Materials

The following are available online at https://www.mdpi.com/2075-4450/11/11/803/s1, Table S1. Proposed Japanese names for *Pterostichus* species examined in this study.
