# Supplementary material for: Unexpected Species Identities and Interspecific Relationships in a Subterranean Beetle Lineage, the Pterostichus macrogenys Species Group (Coleoptera, Carabidae), Revealed by Fine-Scale Field Sampling and Detailed Morphological Comparisons
Source: Insects. 2020 Nov 14;11(11):803. doi: 10.3390/insects11110803 (PMC7698068; doi:10.3390/insects11110803)
Supplement: Supplementary file 1 [file insects-11-00803-s001.pdf]

Table S1. Proposed Japanese name for species treated in the present study

**Species**	**Japanese Name**
P. chokaisanus	Chôkai-ôzu-nagagomimushi
P. kurikomasanus	Kurikoma-ôzu-nagagomim
P. falcispinus	Ishikiri-ôzu-nagagomimushi
P. takadateyamanus	Takadate-ôzu-nagagomimushi
P. adatarasanus	Adatara-ôzu-nagagomimushi
P. gassanus	Gassan-ôzu-nagagomimushi
P. iwakiensis	Iwaki-ôzu-nagagomimushi
P. eboshiyamanus	Eboshi-ôzu-nagagomimushi
P. monolineatus	Marumori-ôzu-nagagomimushi
